# *Oogamochlamys kurilensis* sp. nov. (Chlorophyta, Volvocales) from the Soils of Iturup Island (Sakhalin Region, Russia)

**DOI:** 10.3390/plants12193350

**Published:** 2023-09-22

**Authors:** Vyacheslav Yu. Nikulin, Arthur Yu. Nikulin, Andrey A. Gontcharov, Veronika B. Bagmet, Shamil R. Abdullin

**Affiliations:** Federal Scientific Center of the East Asia Terrestrial Biodiversity, Far Eastern Branch of the Russian Academy of Sciences, 159, 100-Letia Vladivostoka Prospect, Vladivostok 690022, Russia; gontcharov@biosoil.ru (A.A.G.); chara1989@yandex.ru (V.B.B.); crplant@mail.ru (S.R.A.)

**Keywords:** biflagellate green alga, *Oogamochlamys*, new species, SSU rDNA, ITS rDNA secondary structure, morphological characteristics, life cycle, temperate monsoon climate zone

## Abstract

A strain of oogamous biflagellate green alga was isolated during a study on soil algal diversity in the Russian Far East (Sakhalin Region, Iturup Island) and examined using an integrative approach. Phylogenetic analyses, based on the SSU rDNA gene, resolved the new strain as a part of the RL clade (*sensu* Watanabe and Nakada) within Oogamochlamydinia (Volvocales, Chlorophyceae). The strain was similar to members of the genus *Oogamochlamys* (parietal and massive cup-shaped chloroplasts; two apical contractile vacuoles), but was, however, distinguished from them based on the size and shape of the mature vegetative cells, the flagellar length, the presence of only one pyrenoid in both the mature vegetative cells and the zoospores, the anterior nucleus position, and the spermatozoids’ shape. Although a concept of the genus *Oogamochlamys* has been compromised in recent phylogenetic analysis based on the SSU rDNA sequence data and its likely affinity to anisogamous *Chlamydomonas allensworthii*, we described the strain from Iturup Island as *Oogamochlamys kurilensis* sp. nov.

## 1. Introduction

*Chlamydomonas*-like algae (Volvocales/Chlamydomonadales, Chlorophyceae) are green flagellates that typically possess two flagella of equal length and CW flagellar apparatus orientation, a cell wall, and a single chloroplast with pyrenoid(s) [1,2]. Their morphological identification is often difficult due to the small size of the cells, the limited number of taxonomic characters, and their high polymorphism within populations or during the life cycle [3,4]. Chlamydomonadscan be found in almost every aquatic and soil habitat; however, many biodiversity assessment accounts refer only to the genera, and these data require verification. An unambiguous taxonomic affiliation of green flagellates could only be achieved with the establishment of their exact molecular phylogenetic position [5,6].

Simple morphology hides the differentiation of *Chlamydomonas*-like algae and represents a diverse array of lineages within the Volvocales [3,7,8,9,10]. One of these lineages is the Oogamochlamydinia clade, which comprises microalgae that are currently classified in eight genera: *Lobochlamys* T.Pröschold, B.Marin, U.W.Schlösser & M.Melkonian, * Oogamochlamys* Pröschold, B.Marin, U.W.Schlösser & Melkonian [3], *Hapalochloris* Nakada [11], *Rhysamphichloris* Nakada [12], *Gymnomonas* S.Watanabe & T.Nakada [13], *Sarcinochlamys* S.Watanabe [14], *Palmellopsis* Korshikov, *Asterococcus* Scherffel, and a number of species with uncertain taxonomic affiliations.

The genus *Oogamochlamys* comprises three species that were previously members of the genus *Chlamydomonas*: *O. gigantea* (O.Dill) Pröschold, B.Marin, U.W.Schlösser & Melkonian (*C. gigantea*, *C. megalis* H.W.Bischoff & Bold, and *C. capensis* Pocock), *O. ettlii* Pröschold, B.Marin, U.W.Schlösser & Melkonian (*C. gigantea* O.Dill), and *O. zimbabwiensis* (Heimke & R.C.Starr) Pröschold, B.Marin, U.W.Schlösser & Melkonian (*C. zimbabwiensis* Heimke & R.C.Starr). These species are characterized by chloroplast morphology (parietal, massive plastids with ridges on the surface), multiple irregularly distributed pyrenoids, and homothallic protandric oogamy [3,5]. They are mostly found in soil, although some species were also found in bottom sediments [3].

Information on the soil microalgal diversity of the Kuril Islands (Russia) is very scarce and mostly based on traditional approaches [15,16]. During a study on algae in the soils of Iturup Island (Sakhalin Region, Russia), we isolated a strain of *Chlamydomonas*-like green alga and studied it using an integrative approach. SSU rDNA gene sequence comparisons suggested its affinity to *Oogamochlamys* (Oogamochlamydinia clade). The combination of the phylogenetic and morphotaxonomic features of the strain led us to describe the alga from Iturup Island as a new species, *Oogamochlamys kurilensis* sp. nov.

## 2. Results

### 2.1. Taxonomic Treatment

**Figure 1:**
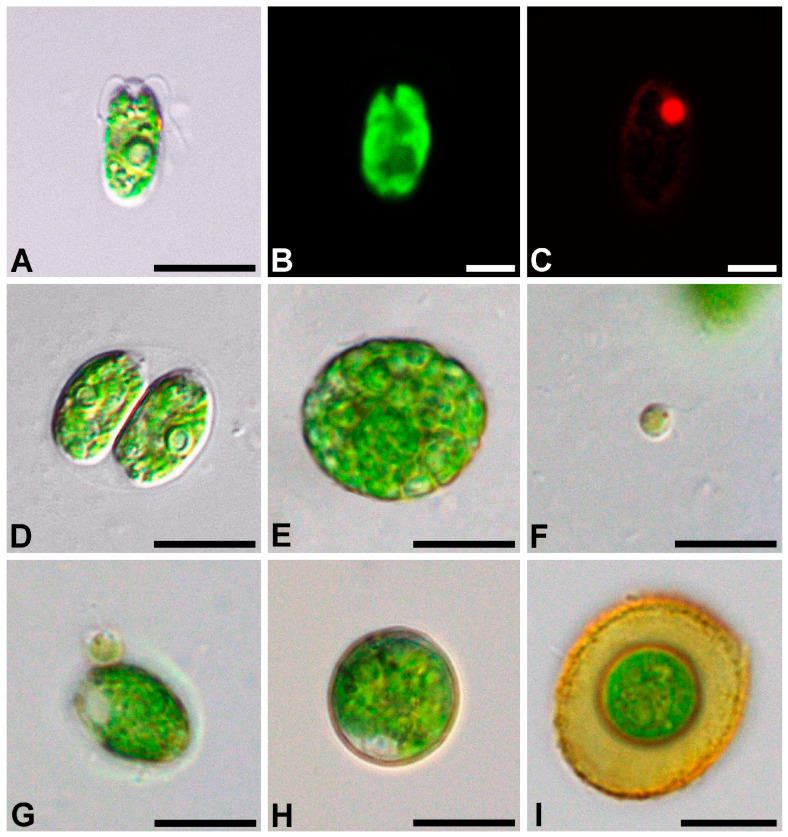
Light micrographs of general morphology (**A**,**D**–**I**), confocal reconstruction of chloroplast morphology (**B**), and confocal optical section of the nucleus (**C**) with a bright-field image-merged fluorescence channel in a *Oogamochlamys kurilensis*, cell with a nucleus stained with DAPI; (**A**–**C**) vegetative cell; (**D**) zoosporangium; (**E**) gametangium; (**F**) spermatozoid; (**G**) spermatozoid attached to a vegetative cell; (**H**) zygote; (**I**) mature zygote. Scale bars: **A**, **D**, **E**, **F**, **G**, **H**, **I** = 10 µm; **B**, **C** = 5 µm.

*Oogamochlamys kurilensis* V.Yu. Nikulin, Sh.R. Abdullin, V.B. Bagmet, A.Yu. Nikulin & A.A. Gontcharov, sp. nov. are shown in Figure 1A–I.

Diagnosis: the cells are ellipsoid to oviform, 6.1–17.1 × 4.5–9.5 μm; the cell wall is thin, with two flagella that are about half the cell length; the papilla broad, rounded. The chloroplast is cup-shaped, parietal, and massive; the chloroplast surface with fine ridges mostly parallel to the cell axis, with one pyrenoid in the lateral position; the eyespotis pale red, elliptic to narrowly elongate in an anterior position; two apical contractile vacuoles, nucleus in the anterior position.

Asexual reproduction by two or four zoospores, nearly spherical to oviform, with one pyrenoid, 5.7–12.4 × 4.0–8.4 μm. The sporangial wall is partially lysing before release of zoospores.

Sexual reproduction occurs via oogamy, homothallic, proterandric; 32–128 spermatozoids are formed in the gametangium; the spermatozoids are 4.0–4.5 × 3.4–4.0 μm, spherical, without a cell wall, and with two flagella nearly 1.5 times longer than the cell; the chloroplast is pale green, with a distinct eyespot, and without a pyrenoid.

The zygotes are not ornamented, are green to brownish-red, and 13.7–22.4 μm in diameter.

Habitat: soil.

Type locality: Russia, Sakhalin Region, Iturup Island (45°09′36″ N, 147°46′37.2″ E), in forest soil under the plant communities with *Sasa kurilensis* (Rupr.) Makino & Shibata, *Quercus crispula* Blume, and *Acer tschonoskii* Maxim.

Etymology: the species is named after the Kuril Islands.

Holotype (designated herein): exsiccatum number VLA-CA-1065; a dried biomass of a unialgal population deposited in the Herbarium, Federal Scientific Center of East Asian Terrestrial Biodiversity, Vladivostok, Russia. Gene sequence: the DNA sequence was obtained from a clonal strain of *Oogamochlamys kurilensis* deposited in GenBank under accession no. OM949811.

Authentic strain: *Oogamochlamys kurilensis* strain VCA-206 was deposited in the Culture Collection of the Laboratory of Botany, Federal Scientific Center of East Asian Terrestrial Biodiversity, Russia.

### 2.2. Phylogenetic Analyses

Results of the BLAST searches revealed that the sequence of the SSU rDNA gene in our strain was highly similar to those in three accessions of *Chlamydomonas* sp. (98–99%) and *Oogamochlamys ettlii* UTEX 2218 (98.31%). When only the ITS region was compared, the similarity to the closest match, *Chlamydomonas allensworthii* R.C.Starr, F.Marner & Jaenicke accessions, was just above 84%.

**Figure 2:**
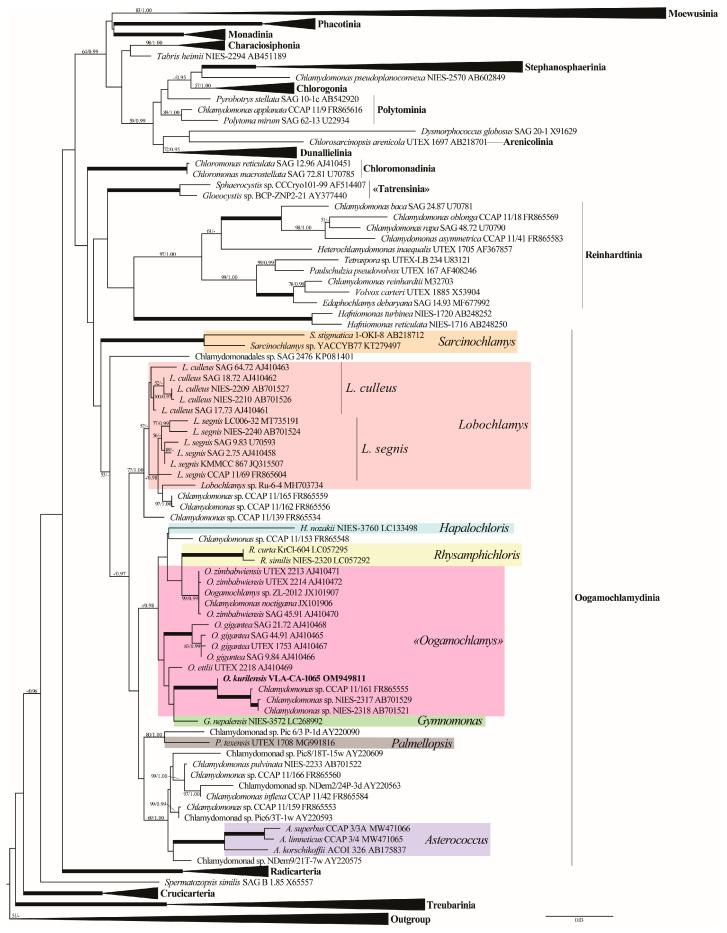
ML phylogenetic tree of the Volvocales (model GTR + I + G) showing the position of the new strain based on the SSU rDNA sequence data (155 sequences, 1695 aligned positions). Some clades are collapsed. Supports [(BP) > 50% and (PP) > 0.95: ML/BI] are provided above/below the branches. The new strain and branches with 100% BP and 1.00 PP are shown in boldface. The major clade designations follow Nakada et al. [9].

Phylogenetic analyses of 155 SSU rDNA sequences representing major groups of the Volvocales (*sensu* Nakada et al. [9]) placed the new strain as a member of the Oogamochlamydinia clade that was resolved only topologically (Figure 2 and Figure S1). This lineage included the genera *Asterococcus*, *Sarcinochlamys*, *Gymnomonas*, *Hapalochloris*, *Lobochlamys*, *Oogamochlamys*, *Palmellopsis*, *Rhysamphichloris*, and numerous *Chlamydomonas* representatives. The overall phylogenetic resolution in the Oogamochlamydinia was weak. Four out of five genera represented by two or more accessions, *Asterococcus*, *Sarcinochlamys*, *Lobochlamys*, and *Rhysamphichloris*, attained moderate to strong support.

Our species was placed in a clade (−/0.98) that comprised representatives of the genera *Oogamochlamys*, *Rhysamphichloris*, *Gymnomonas*, and *Hapalochloris,* as well as four *Chlamydomonas* sp. strains. *Oogamochlamys* was not monophyletic and split into three species lineages: *O. gigantea* (100/1.00)*, O. zimbabwiensis* (99/0.99)*,* and *O. ettlii*. *Oogamochlamys kurilensis* was a sister (100/1.00) to a robust clade uniting *Chlamydomonas* sp. CCAP 11/161, NIES-2317, and NIES-2318.

**Figure 3:**
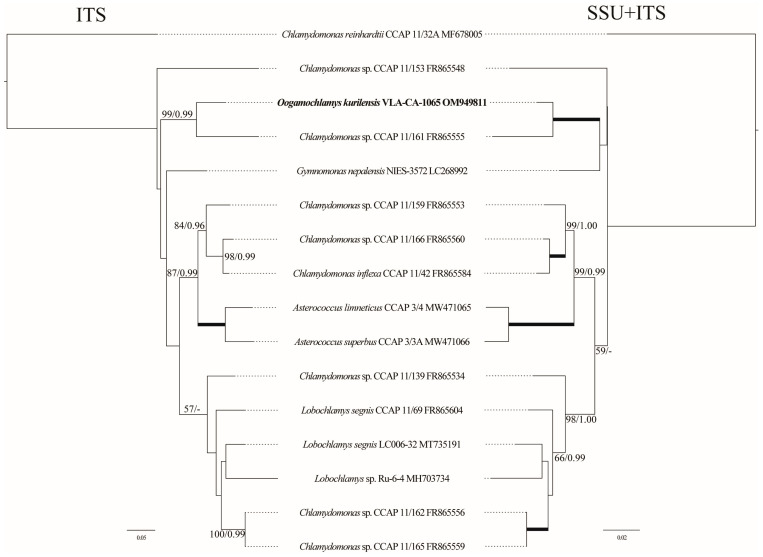
Tanglegram (ML) representing the position of the new strain based on the ITS rDNA sequence data (703 aligned positions of 16 sequences) and the SSU+ITS rDNA dataset (2438 aligned positions). See Figure 2 legend for details.

To access the phylogenetic affinity of *O. kurilensis* with a more variable and likely informative marker, we assembled a dataset using sequences of the ITS region, and analyzed it separately and in a concatenation with the SSU rDNA data for the same strains (Figure 3). However, ITS data were only available in the GenBank for 15 out of the 50 Oogamochlamydinia accessions, which significantly limited the taxon sampling in this lineage. The topologies of the ITS and SSU+ITS rDNA trees were generally consistent with the SSU-based tree and confirmed the close relationship between *O. kurilensis* and *Chlamydomonas* sp. CCAP 11/161. Unfortunately, no ITS sequences were available for other *Oogamochlamys* species; therefore, these analyses could not clarify the genus concept and relationships between *O. kurilensis* and other species.

**Figure 4:**
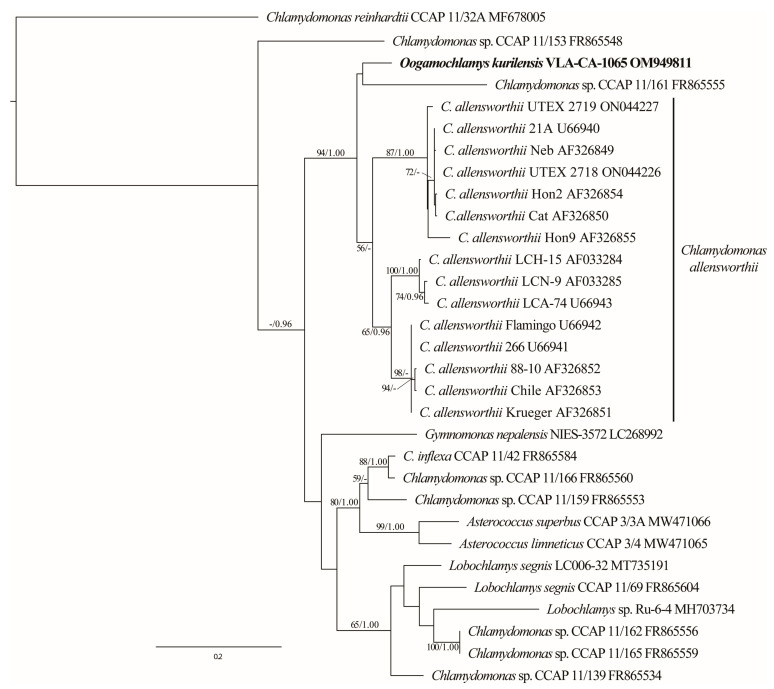
ML phylogenetic tree (GTR + I + G model) based on ITS rDNA sequence data (540 aligned positions of 31 sequences) showing the close relationship between *O. kurilensis* and *C. allensworthii*. See Figure 2 legend for details.

Since the *C. allensworthii* ITS sequences showed the highest similarity to that of *O. kurilensis* in the BLAST searches (84%), we analyzed the ITS dataset that included 15 accessions of the former species. The *C. allensworthii* clade was a sister (94/1.00) to a lineage comprising *O. kurilensis* and *Chlamydomonas* sp. CCAP 11/161 (Figure 4).

For the comparison of the ITS2 secondary structures between the closely related sequences, we have reconstructed the ITS2 secondary structure of *O. kurilensis*. Figure S2 illustrates the proposed base pairing. According to our predictions, the spacer was characterized by a typical structure with four helices and five single-stranded domains. Approximately 90% of the nucleotides were involved in the formation of the helicies in ITS2.

**Figure 5:**
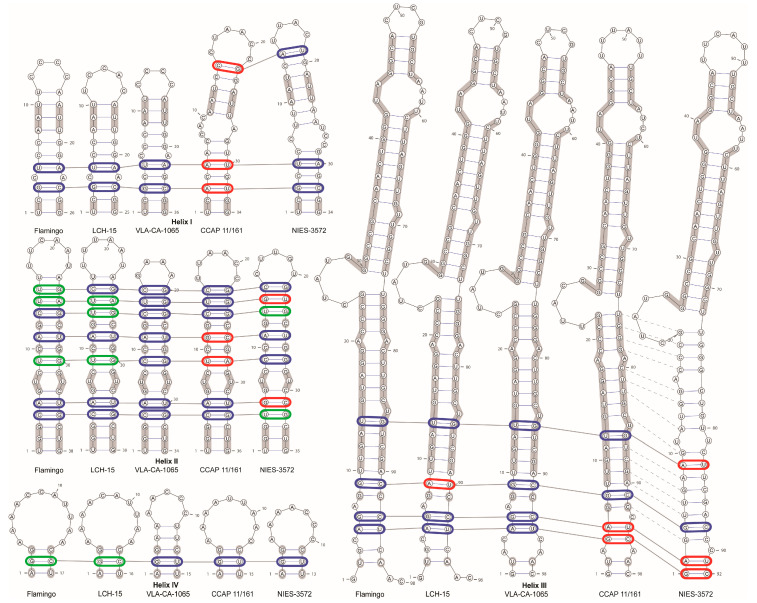
ITS2 secondary structure models for the new and related strains based on Mfold predictions. (*C. allensworthii* Flamingo U66942, *C. allensworthii* LCH-15 AF033284, *O. kurilensis* VLA-CA-1065 OM949811, *Chlamydomonas* sp. CCAP 11/161 FR865555, *Gymnomonas nepalensis* NIES-3572 LC268992). Compensatory (CBCs) and hemi-compensatory base changes (hCBCs) are shown in red and green colors, respectively. Identical bases are highlighted with a thick grey line. Homologous base pairs of helix III are indicated by dashed lines.

The comparison of the ITS2 secondary structures between *O. kurilensis* (Figure 5) and its closely related sequences (*C. allensworthii* Flamingo U66942, *C. allensworthii* LCH-15 AF033284, *Chlamydomonas* sp. CCAP 11/161, *Gymnomonas nepalensis* NIES-3572) showed that these algae have similar patterns of bulges and terminal loops (Figure 5). The helix I in *O. kurilensis* and *C. allensworthii* was shorter than those in other sequences, and the helix IV in *O. kurilensis* differed from others in terms of a longer helical part and shorter terminal loop (five pairs and five bases vs. three pairs and seven to eleven bases). We found eleven compensatory base changes (CBCs) and six hemi-compensatory base changes (hCBCs; Figure 5) in every helical domain. *O. kurilensis* differed from the most similar *C. allensworthii* in three hCBCs in the ITS2 (Helix II: U-G → U-A at positions 14–25 and C-G → U-G at positions 9–30; Helix IV: G-U → G-C, at position 2–14; Figure 5). The specific CBC and hCBC patterns in helices II and III may indicate that *O. kurilensis* and the American strain (CCAP 11/161) are different species of the same genus *Oogamochlamys*.

## 3. Discussion

In the present study, a novel *Chlamydomonas*-like biflagellated green alga, named *Oogamochlamys kurilensis* sp. nov., was described from the soil of Iturup Island (Russia) based on an integrative approach. SSU rDNA sequence comparisons (Figure 2 and Figure S1) confidently placed this species in the Oogamochlamydinia clade. The gene sequence data were not conclusive in resolving the generic affiliation of the new species by placing it into a weakly supported clade that included representatives of four genera and four unnamed *Chlamydomonas* strains (Figure 2). Moreover, the most sampled in this clade genus *Oogamochlamys* was resolved paraphyletic. The non-monophyly of *Oogamochlamys* in our analyses contradicts earlier studies based on a limited taxon sampling [3,14] but agrees with the later, more inclusive phylogenies [9,11,12]. The lack of support for *Oogamochlamys* was likely due to a low (1–3%) SSU rDNA sequence divergence between its species. The absence of ITS rDNA data for representatives of the *Oogamochlamys* species precluded the application of this more variable marker to confirm the affinity of *O. kurilensis* to the genus.

Nevertheless, we can conclude that the new species is a member of the RL clade within Oogamochlamydinia [13]. This polygeneric lineage is characterized by a wide range of morphologies that were used to delineate genera and species, and we mostly rely on phenotypic features in assigning the strain from Kurils. The two wall-less genera, *Hapalochloris* and *Gymnomonas*, could be excluded from consideration because of their distinct feature [11,13]. The remaining *Rhysamphichloris* and *Oogamochlamys* are similar in terms of their presence of several pyrenoids in the cell, but somewhat differ in their chloroplast morphology, which are *Amphichloris*-type with anterior and posterior thickenings, and deep-cup-shaped, respectively [3,11]. The morphology of the chloroplasts described in *O. kurilensis* (cup-shaped, with one basally positioned pyrenoid) does not fit the diagnosis of both genera but is similar to that in the wall-less *Hapalochloris* and *Gymnomonas*.

Chloroplast morphology and the number of pyrenoids are polymorphic features, and could hardly be used to differentiate volvocalean genera. In contrast to that, the type of sexual reproduction is believed to be a good generic character for *Chlamydomonas*-like algae [3,5]. However, in the Oogamochlamydinia, like many other lineages, no sexual reproduction was observed in six out of the eight genera of which it is comprised. Namely, *Lobochlamys* was characterized by isogamous, homo- or heterothallic sexual reproduction, and *Oogamochlamys* by homothallic proterandric oogamy [3]. The new species shares the latter mode of sexual reproduction with other *Oogamochlamys* species; therefore, we assigned *O. kurilensis* to this genus.

It should be noted that a likely paraphyly of the *Oogamochlamys* clade that was resolved in recent SSU rDNA-based analyses, as well as the close relationship between oogamous *O. kurilensis* and anizogamous *C. allensworthii* suggested in the ITS rDNA data (Figure 4), questions the concept of the genus *Oogamochlamys*. Apparently, the unresolved relationship between four genera and additional lineages in the in the RL clade [13] require further scrutiny to define generic boundaries. If confirmed, the alliance of *O. kurilensis* and *C. allensworthii* may indicate a diversity of sexual reproduction modes in *Oogamochlamys* that requires a diagnosis adjustment or erection of a new genus.

Some phenotypic features of our taxon correspond to the diagnoses of other members of the genus *Oogamochlamys* (e.g., the shape of the papilla, and the number and distribution of contractile vacuoles in *O. zimbabwiensis*; the chloroplast surface with fine ridges in *O. ettlii*; the number of spermatozoids in the gametangium in *O. gigantea*; Table 1) but their combination clearly differentiates *O. kurilensis*. Moreover, the size of the mature vegetative cells and the flagellar length of this species are the smallest in the genus. The shape of the mature vegetative cells of *O. kurilensis* (oval to cylindrical–oviform) is also not typical for *Oogamochlamys*. The most noticeable morphological differences between the new species and the rest of the genus is the presence of a single pyrenoid in both the mature vegetative cells and the zoospores, while in most other species of *Oogamochlamys*, the number of pyrenoids varies from two to twenty (Table 1). It was shown that the presence of pyrenoids in *Chlorogonium* depends on the culture conditions (autotrophic or heterotrophic; [17]), but our strain was examined under the same autotrophic conditions as other accessions of *Oogamochlamys* studied by Pröschold et al. [3] and no variation in the number of pyrenoids was detected.

The composition and structure of the *Oogamochlamys* lineage (Figure 2, Figure 3 and Figure 4) suggest the possible presence of a number of undescribed species in the genus. These are *Chlamydomonas* sp. CCAP 11/161 and two highly similar strains from Japan (NIES-2317 and NIES-2318) that comprised a clade with *O. kurilensis*. Based on the CCAP 11/161 images (https://www.ccap.ac.uk/catalogue/strain-11-161?mfp=8-genus-name%5BChlamydomonas%5D&limit=100; accessed on 2 June 2023), we can conclude that this alga is similar to our strain in terms of the cell morphology. In addition to that, another related morpho-species, *C. allensworthii,* likely includes at least five biological species [5].

*Oogamochlamys* representatives are reported relatively unfrequently in biodiversity assessments and these records likely suggest their somewhat restricted distribution in nature. Three species have previously been recorded in southern Africa (*O. gigantea* and *O. zimbabwiensis* in South Africa and Zimbabwe; *O. ettlii* only in Zimbabwe). In addition, *O. gigantea* was found in North America (USA, California, and Texas; [3]; Table 2) as well. Until recently, no *Oogamochlamys* species were known from Asia, but the finding of *O. zimbabwiensis* (strain ZL-2012) in Korea (Figure 2) and the new species *O. kurilensis* in the Kuril Islands (Russia, Sakhalin Region) extends the genus distribution range. *Chlamydomonas* strains from Japan (NIES-2317 and NIES-2318) that likely represent yet undescribed members of the genus that are closely related to *O. kurilensis* were also collected in Asia. *Chlamydomonas allensworthii,* also showing affinity to *Oogamochlamys,* was found in most continents except Asia and Antarctica [18,19]. All these taxa were found in areas with temperate climate conditions and their distribution ranges in the southern and northern hemispheres are confined to approximately the same latitudes (Table 2).

The species could be tentatively divided into three ecological groups: soil inhabitants (*O. ettlii*, *O. kurilensis*, *C. allensworthii*), soil—benthic (*O. gigantea*, *O. zimbabwiensis*, *Chlamydomonas* sp. (NIES-2317, NIES-2318)), and aquatic (*Chlamydomonas* sp. CCAP 11/161). Thus, they occur in soil and aquatic environments but are not eurybiontic.

Most likely, the small number of sequences of *Oogamochlamys* indicate their rare occurrence or difficulties in cultivating and identification. Further studies on the ITS structures and ultrastructural features are required for the more complete comparison of *Oogamochlamys* species and related strains.

## 4. Materials and Methods

### 4.1. Study Site, Culture Conditions and Light Microscopy

Iturup is the largest island in the Kuril Island arch and belongs to the Southern Kurils. There are 20 volcanoes on Iturup Island; nine of these are active. The climate here is temperate maritime, and is formed under the influence of the currents of the Sea of Okhotsk and the Pacific Ocean, as well as being affected by monsoon activity ([20]; according to Köppen [21]—warm-summer, humid, continental climate (Dfb)). Cambisols, often with high humus content (up to 30% in the upper horizon), is the typical soil type for a major part of Iturup Island [22,23].

A soil sample was collected under the plant communities with *Sasa kurilensis* (Rupr.) Makino & Shibata, *Quercus crispula* Blume, *Acer tschonoskii* Maxim on Iturup Island (Sakhalin Region, Russia; 45°09′36″ N, 147°46′37.2″ E) on 3 August 2018. The sampling was carried out using standard methods [24]. A strain of flagellated green algae was isolated from this sample using the micro-pipette method [25], and was cultured in liquid nutrient medium Waris-H [26] at 20–22 °C with a photon fluence of 17.9–21.4 μmol photons·m^−2^s^−1^ in a 16:8 h light/dark cycle. The strain was maintained in the culture collection of the Laboratory of Botany in the Federal Scientific Center of East Asian Terrestrial Biodiversity, Russian Federation (strain number VCA-206).

The morphology of the vegetative and reproductive cells was examined using an Olympus BX 53 light microscope (Olympus Corporation, Tokyo, Japan) equipped with Nomarski DIC optics and an Olympus DP27 digital camera (Olympus Corporation, Tokyo, Japan). The cultures were repeatedly examined throughout their life cycle stages, i.e., in cultures of different ages after transfer.

For the confocal laser scanning microscopy, 0.01% Triton X-100 was added to the culture of living algal cells to increase their membrane permeability. Then, the cells were fixed in FAA (3.7%: formaldehyde: 50% ethanol: 5% acetic acid) for 20 min, then rinsed twice and counterstained with DAPI (4,6-diamidino-2-phenylindole, Molecular Probes Inc., Eugene, OR, USA) at a final concentration of 5 µg/mL. After another rinse of the samples, fluorescence was detected with an LSM 710 LIVE confocal laser scanning microscope (Carl Zeiss, Oberkochen, Germany) at the Instrumental Centre of Biotechnology and Gene Engineering of FSCEATB FEB RAS. DAPI fluorescence was detected at 410–497 nm and the autofluorescence of the chloroplasts was recorded in the additional emission channel after 600 nm using the Plan-Apochromat 63x/1.40 Oil DIC M27 objective with digital zoom. Three-dimensional files of the captured images were recorded and analyzed using ZEN microscope software.

### 4.2. DNA Extraction, Amplification, and Sequencing

For the DNA analysis, the cultures were harvested during the exponential growth phase and concentrated via centrifugation. Total genomic DNA was extracted, as described previously by Abdullin et al. [27]. For the amplification of the SSU rDNA gene and ITS region, the following primers were used: 82F (5′-GAAACTGCGAATGGCTC-3′; [28]), ITS4R (5′-TCCTCCGCTTATTGATATGC-3′; [29]). PCR was performed using an Encyclo Plus PCR kit (Evrogen, Moscow, Russia) with a T100 Thermal Cycler (Bio-Rad Laboratories, Inc., Hercules, CA, USA) and the parameters described by Mikhailyuk et al. [30]. The PCR products were purified using an ExoSAP-IT PCR Product Cleanup Reagent (Affymetrix Inc., Santa Clara, CA, USA) and sequenced in both directions using an ABI 3500 genetic analyzer (Applied Biosystems, Waltham, MA, USA) with a BigDye terminator v.3.1 sequencing kit (Applied Biosystems, Waltham, MA, USA), and the same primers were used for the PCR, plus SSU528F-800 (5′-CGGTAATTCCAGCTCC-3′; [31]), 920F (5′-GAAACTTAAAKGAATTG-3′; [32]), n1400R (5′-GGTAGGAGCGACGGGCGGTGTGTAC-3′; [33]), and Bd18SF1 (5′-TTTGTACACACCGCCCGTCGC-3′; [34]). Sequences were assembled with the Staden Package v.1.4 [35]. The contig sequence covering the partial SSU rDNA and the complete ITS region was deposited in GenBank under accession number OM949811.

### 4.3. Alignment, Secondary Structure Modeling, and Datasets

In order to clarify the phylogenetic position of the new strain, four datasets were used: (i) the SSU rDNA alignment, based on the dataset of Nakada et al. [9], including 155 taxa and 1695 bp of representatives of the Volvocales and Sphaeropleales, used as an outgroup; (ii) the concatenated dataset of 16 SSU and ITS rDNA sequences (2438 bp); (iii) the ITS rDNA dataset of 16 sequences (703 bp) of the Oogamochlamydinia clade and its sister lineage, Reinhardtinia, used as an outgroup; (iv) the ITS rDNA dataset of 31 sequences (540 bp) of the Oogamochlamydinia clade and *C. allensworthii,* with the Reinhardtinia used as an outgroup. The datasets were enriched by all those accessions showing an ultimate similarity to the sequences gained from our strain, as inferred from the BLAST searches (https://blast.ncbi.nlm.nih.gov/Blast.cgi; accessed on 10 January 2023). The sequences were aligned manually in the SeaView program [36].

The Mfold web server (http://www.unafold.org/mfold/applications/rna-folding-form.php; accessed on 20 January 2023; [37]) was used with the default settings to generate the ITS2 rRNA secondary structures, which were then visualized using the program VARNA [38]. An ITS2 model was constructed based on the models of *C. reinhardtii* and *C. allensworthii* proposed by Pröschold et al. [39] and Coleman et al. [19], respectively.

### 4.4. Phylogenetic Analysis

Maximum likelihood (ML) analysis was carried out using PAUP 4.0b10 [40]. Bayesian inference (BI) was performed using MrBayes 3.1.2 [41]. In order to determine the most appropriate DNA substitution model for the datasets, the Akaike information criterion (AIC; [42]) was applied with jModelTest 2.1.1 [43]. The GTR + I + G, SYM + I + G and their combination (partition) models were selected as the best fits for our SSU, ITS rDNA, and concatenated datasets, respectively. The ML analysis was done using heuristic searches with a branch-swapping algorithm (tree bisection and reconnection). In the BI, two parallel MCMC runs were carried out for 5 million and 300 thousand generations, sampling every 100 generations for a total of 50,000, 3000, and 3000 samples for the SSU, ITS rDNA, and concatenated datasets, respectively. The convergence of the two chains was assessed, and the stationarity was determined according to the ‘sump’ plot, with the first 25% of samples discarded as a burn-in. The convergence of the stationary distribution was accessed using the ESS values (>200) using Tracer v.1.7.1 [44]. The robustness of the ML trees was estimated using bootstrap percentages (BP; [45]) and posterior probabilities (PP) in the BI. A BP < 50% and PP < 0.95 were not considered. The ML-based bootstrap analysis was inferred using the web service RAxML v.7.7.1 (http://embnet.vital-it.ch/raxml-bb/; accessed on 2 February 2023; [46]).

## Figures and Tables

**Table 1:** Comparison of features characterized *Oogamochlamys* genus members, including *C. allensworthii* and the new species, *O. kurilensis*.

Character	*O. gigantea*	*O. ettlii*	*O. zimbabwiensis*	*O. kurilensis* sp. nov.	*C. allensworthii*
Mature vegetative cells					
shape	broadly rounded–cylindrical–oviform			oval–cylindrical–oviform	ellipsoidal to almost spherical
size, μm	30–50 × 25–35	16–27 × 18–22	15–22 × 15–20	6.1–17.1 × 4.5–9.5	15 × 11
Cell wall	thin				
Cell wall papilla	small, rounded		broad, rounded, two humped or absent	broad, rounded	absent
Flagellar length	about as long as the cell			about half of the cell	about as long as the cell
Chloroplast shape	cup-shaped parietal, massive, surface with coarse ridges	cup-shaped, parietal, massive, surface with fine ridges	cup-shaped, parietal, massive, surface with very fine ridges	cup-shaped, parietal, massive, surface with fine ridges	cup-shaped
Pyrenoids	10–16 (–20)	3–8	2–6	1	1–3
shape	discontinuous, matrix multipartite				nd
Eyespot color	pale red				nd
shape	elliptic to narrowly elongated		elliptic to narrowly elongated or punctiform	elliptic to narrowly elongated	elliptic
position in the chloroplast	anterior				
Contractile vacuoles	2 apical + many (>20), distributed over the whole cell surface		2 apical	2 apical	4 apical
Nucleus position	central or slightly anterior			anterior	nd
Zoospores	4 (2–8)			2–4	2–8
shape	nearly spherical			nearly spherical–oviform	nd
pyrenoids	4–8	3–6	2–4	1	nd
size, μm	10–15	10–12		5.7–12.4 × 4.0–8.4	nd
Sexual reproduction	oogamy, homothallic, proterandric				anisogamy
Spermatozoids	32–64 (rarely 128)	16		32–128	nd
shape	teardrop-shaped, without cell wall, with two flagella nearly 1.5 times as long as the cell, with two apical contractile vacuoles, chloroplast reduced, pale green, with a distinct eyespot, without pyrenoid			round-shaped, without cell wall, with two flagella nearly 1.5 times as long as the cell, chloroplast reduced, pale green, with a distinct eyespot, without pyrenoid	teardrop-shaped
size, μm	6–10 × 4–6	4–6 × 3–5		4.0–4.5 × 3.4–4.0	6 × 4 and larger
Zygote	ornamented with regular, flat-stopped projections, green to brownish-red	not ornamented, green		not ornamented, green to brownish-red, diameter 13.7–22.4 μm	with a broad, hyaline wall at first, with crenulations forming on the surface later
References	[3]			This study	[18]

Notes: nd—no data.

**Table 2:** Comparison of climate conditions and habitats of members and potential representatives of the *Oogamochlamys* genus.

Species	Geography	Climate Conditions (Köppen 1936)	Habitat	Strain
*O. gigantea*	Africa (South Africa)	Warm—summer, Mediterranean (Csb)	soil	SAG 44.91
	Africa (Zimbabwe)	nd	soil	SAG 22.98
	North America (USA, California)	Warm—summer, Mediterranean (Csb)	pond soil	SAG 21.72
	North America (USA, Texas)	humid subtropical (Cfa)	soil	SAG 9.84
*O. zimbabwiensis*	Africa (South Africa)	Warm—summer, Mediterranean (Csb)	soil	SAG 45.91
	Africa (Zimbabwe)	nd	soil	UTEX LB 2214
	Africa (Zimbabwe)	nd	soil	SAG 2316
*Oogamochlamys* sp. (putative, *O. zimbabwiensis*)	Asia (Republic of Korea)	Humid, continental (Dwa)	bottom sediment	ZL-2012
*O. ettlii*	Africa (Zimbabwe)	nd	soil	UTEX 2218
*O. kurilensis*	Asia (Russia, Sakhalin Region, Iturup Island)	Warm—summer, humid, continental (Dfb)	soil	VCA-206
*Chlamydomonas* sp.	Asia (Japan, Saitama)	Humid, subtropical (Cfa)	freshwater (paddy soil)	NIES-2317, NIES-2318
*Chlamydomonas* sp.	North America (USA, Minnesota)	Warm—summer, humid, continental (Dfb)	freshwater	CCAP 11/161
*C. allensworthii*	North America (USA, California)	warm—summer, Mediterranean (Csb)	soil	LCH-15, LCN-9, LCA-74
	North America (USA, Texas)	Humid, subtropical (Cfa)	nd	21A, 266
	Europe (Germany, Koln)	temperate—oceanic (Cfb)	nd	Flamingo
	North America (USA, Nebraska)	Humid, continental (Dfa)	nd	isolate 1 (Neb)
	North America (USA, Texas)	Hot, semi-arid (BSh)	soil	isolate 2 (Cat)
	Africa (South Africa)	Hot, semi-arid (BSh)	nd	isolate 3 (Krueger)
	Australia (Lismore)	Humid, subtropical (Cfa)	nd	isolate 4 (88-10)
	South America (Chile, Lago Cisne)	subpolar variety of the oceanic (Cfc)	nd	isolate 5 (Chile)
	Oceania (USA, Hawaii)	Hot, semi-arid (BSh)	nd	isolate 6 (Hon2), isolate 7 (Hon9)
	North America (USA, California)	warm—summer, Mediterranean (Csb)	soil	UTEX 2718
	North America (USA, Texas)	Hot, semi-arid (BSh)	soil	UTEX 2719

Notes: nd—no data.

## Data Availability

The data presented in this study are available on request from the corresponding author. In addition, the data that support the findings of this study are openly available in GenBank.

## Supplementary Materials

The following supporting information can be downloaded at: https://www.mdpi.com/article/10.3390/plants12193350/s1, Figure S1: ML phylogenetic tree of the Volvocales (model GTR + I + G) showing position of the new strain based on SSU rDNA sequence data (1695 aligned positions of 155 sequences) with expanding of collapsed clades. See Figure 2 legend for details. Figure S2: ITS2 rRNA secondary structure model for *Oogamochlamys kurilensis* (VLA-CA-1065, OM949811) based on Mfold predictions.
