# Supplementary material for: Oogamochlamys kurilensis sp. nov. (Chlorophyta, Volvocales) from the Soils of Iturup Island (Sakhalin Region, Russia)
Source: Plants (Basel). 2023 Sep 22;12(19):3350. doi: 10.3390/plants12193350 (PMC10574126; doi:10.3390/plants12193350)
Supplement: Supplementary file 1 [file plants-12-03350-s001.zip › Figure S1.pdf]

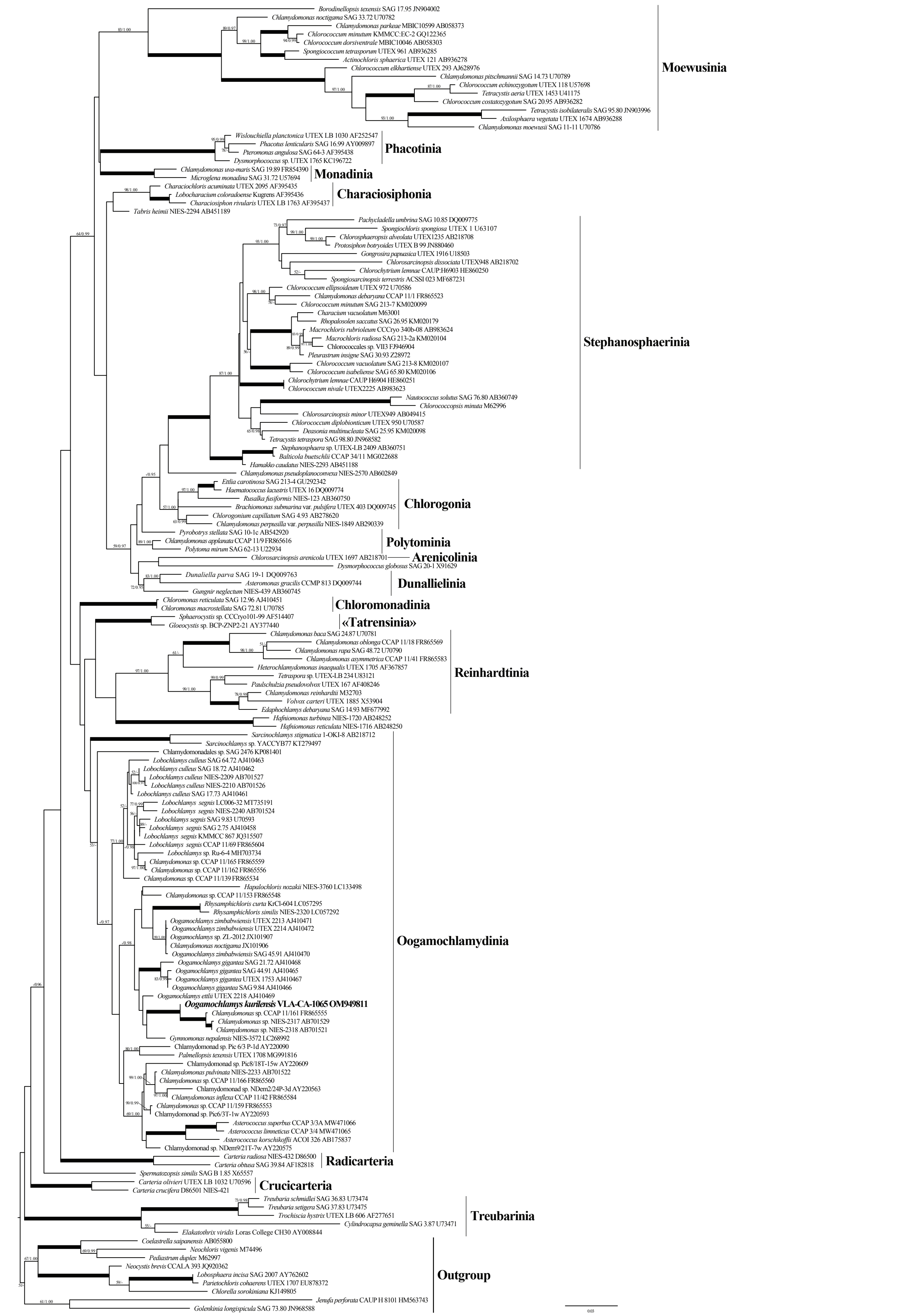
Borodinellopsis texensis SAG 17.95 JN904002
Chlamydomonas noctigama SAG 33.72 U70782
Chlamydomonas parkeae MBIC10599 AB058373
Chlorococcum minutum KMMCC:EC-2 GQ122365
Chlorococcum dorsiventrale MBIC10046 AB058303
Spongiococcum tetrasporum UTEX 961 AB936285
Actinochloris sphaerica UTEX 121 AB936278
Chlorococcum elkhartiense UTEX 293 AJ628976
Chlamydomonas pitschmannii SAG 14.73 U70789
Chlorococcum echinozygotum UTEX 118 U57698
Tetracystis aeria UTEX 1453 U41175
Chlorococcum costatozygotum SAG 20.95 AB936282
Tetracystis isobilateralis SAG 95.80 JN903996
Axilosphaera vegetata UTEX 1674 AB936288
Chlamydomonas moewusii SAG 11-11 U70786
Moewusinia
Wislouchiella planctonica UTEX LB 1030 AF252547
Phacotus lenticularis SAG 16.99 AY009897
Pteromonas angulosa SAG 64-3 AF395438
Dysmorphococcus sp. UTEX 1765 KC196722
Phacotinia
Chlamydomonas uva-maris SAG 19.89 FR854390
Microglena monadina SAG 31.72 U57694
Monadinia
Characiochloris acuminata UTEX 2095 AF395435
Lobocharacium coloradoense Kugrens AF395436
Characiosiphon rivularis UTEX LB 1763 AF395437
Tabris heimii NIES-2294 AB451189
Characiosiphonia
Pachycladella umbrina SAG 10.85 DQ009775
Spongiochloris spongiosa UTEX 1 U63107
Chlorosphaeropsis alveolata UTEX1235 AB218708
Protosiphon botryoides UTEX B 99 JN880460
Gongrosira papuasica UTEX 1916 U18503
Chlorosarcinopsis dissociata UTEX948 AB218702
Chlorochytrium lemnae CAUP:H6903 HE860250
Spongiosarcinopsis terrestris ACSSI 023 MF687231
Chlorococcum ellipsoideum UTEX 972 U70586
Chlamydomonas debaryana CCAP 11/1 FR865523
Chlorococcum minutum SAG 213-7 KM020099
Characium vacuolatum M63001
Rhopalosolen saccatus SAG 26.95 KM020179
Macrochloris rubrioleum CCCryo 340b-08 AB983624
Macrochloris radiosa SAG 213-2a KM020104
Chlorococcales sp. VII3 FJ946904
Pleurastrum insigne SAG 30.93 Z28972
Chlorococcum vacuolatum SAG 213-8 KM020107
Chlorococcum isabeliense SAG 65.80 KM020106
Chlorochytrium lemnae CAUP H6904 HE860251
Chlorococcum nivale UTEX2225 AB983623
Nautococcus solutus SAG 76.80 AB360749
Chlorococcopsis minuta M62996
Chlorosarcinopsis minor UTEX949 AB049415
Chlorococcum diplobionticum UTEX 950 U70587
Deasonia multinucleata SAG 25.95 KM020098
Tetracystis tetraspora SAG 98.80 JN968582
Stephanosphaera sp. UTEX-LB 2409 AB360751
Balticola buetschlii CCAP 34/11 MG022688
Hamakko caudatus NIES-2293 AB451188
Stephanosphaerinia
Chlamydomonas pseudoplanoconvexa NIES-2570 AB602849
Ettlia carotinosa SAG 213-4 GU292342
Haematococcus lacustris UTEX 16 DQ009774
Rusalka fusiformis NIES-123 AB360750
Brachiomonas submarina var. pulsifera UTEX 403 DQ009745
Chlorogonium capillatum SAG 4.93 AB278620
Chlamydomonas perpusilla var. perpusilla NIES-1849 AB290339
Chlorogonia
Pyrobotrys stellata SAG 10-1c AB542920
Chlamydomonas applanata CCAP 11/9 FR865616
Polytoma mirum SAG 62-13 U22934
Polytominia
Chlorosarcinopsis arenicola UTEX 1697 AB218701
Dysmorphococcus globosus SAG 20-1 X91629
Arenicolinia
Dunaliella parva SAG 19-1 DQ009763
Asteromonas gracilis CCMP 813 DQ009744
Gungnir neglectum NIES-439 AB360745
Dunallielinia
Chloromonas reticulata SAG 12.96 AJ410451
Chloromonas macrostellata SAG 72.81 U70785
Chloromonadinia
Sphaerocystis sp. CCCryo101-99 AF514407
Gloeocystis sp. BCP-ZNP2-21 AY377440
«Tatrensinia»
Chlamydomonas baca SAG 24.87 U70781
Chlamydomonas oblonga CCAP 11/18 FR865569
Chlamydomonas rapa SAG 48.72 U70790
Chlamydomonas asymmetrica CCAP 11/41 FR865583
Heterochlamydomonas inaequalis UTEX 1705 AF367857
Tetraspora sp. UTEX-LB 234 U83121
Paulschulzia pseudovolvox UTEX 167 AF408246
Chlamydomonas reinhardtii M32703
Volvox carteri UTEX 1885 X53904
Edaphochlamys debaryana SAG 14.93 MF677992
Hafniomonas turbinea NIES-1720 AB248252
Hafniomonas reticulata NIES-1716 AB248250
Reinhardtinia
Sarcinochlamys stigmatica 1-OKI-8 AB218712
Sarcinochlamys sp. YACCYB77 KT279497
Chlamydomonadales sp. SAG 2476 KP081401
Lobochlamys culleus SAG 64.72 AJ410463
Lobochlamys culleus SAG 18.72 AJ410462
Lobochlamys culleus NIES-2209 AB701527
Lobochlamys culleus NIES-2210 AB701526
Lobochlamys culleus SAG 17.73 AJ410461
Lobochlamys segnis LC006-32 MT735191
Lobochlamys segnis NIES-2240 AB701524
Lobochlamys segnis SAG 9.83 U70593
Lobochlamys segnis SAG 2.75 AJ410458
Lobochlamys segnis KMMCC 867 JQ315507
Lobochlamys segnis CCAP 11/69 FR865604
Lobochlamys sp. Ru-6-4 MH703734
Chlamydomonas sp. CCAP 11/165 FR865559
Chlamydomonas sp. CCAP 11/162 FR865556
Chlamydomonas sp. CCAP 11/139 FR865534
Hapalochloris nozakii NIES-3760 LC133498
Chlamydomonas sp. CCAP 11/153 FR865548
Rhysamphichloris curta KrCl-604 LC057295
Rhysamphichloris similis NIES-2320 LC057292
Oogamochlamys zimbabwiensis UTEX 2213 AJ410471
Oogamochlamys zimbabwiensis UTEX 2214 AJ410472
Oogamochlamys sp. ZL-2012 JX101907
Chlamydomonas noctigama JX101906
Oogamochlamys zimbabwiensis SAG 45.91 AJ410470
Oogamochlamys gigantea SAG 21.72 AJ410468
Oogamochlamys gigantea SAG 44.91 AJ410465
Oogamochlamys gigantea UTEX 1753 AJ410467
Oogamochlamys gigantea SAG 9.84 AJ410466
Oogamochlamys ettlii UTEX 2218 AJ410469
Oogamochlamys kurilensis VLA-CA-1065 OM949811
Chlamydomonas sp. CCAP 11/161 FR865555
Chlamydomonas sp. NIES-2317 AB701529
Chlamydomonas sp. NIES-2318 AB701521
Gymnomonas nepalensis NIES-3572 LC268992
Chlamydomonad sp. Pic 6/3 P-1d AY220090
Palmellopsis texensis UTEX 1708 MG991816
Chlamydomonad sp. Pic8/18T-15w AY220609
Chlamydomonas pulvinata NIES-2233 AB701522
Chlamydomonas sp. CCAP 11/166 FR865560
Chlamydomonad sp. NDem2/24P-3d AY220563
Chlamydomonas inflexa CCAP 11/42 FR865584
Chlamydomonas sp. CCAP 11/159 FR865553
Chlamydomonad sp. Pic6/3T-1w AY220593
Asterococcus superbus CCAP 3/3A MW471066
Asterococcus limneticus CCAP 3/4 MW471065
Asterococcus korschikoffii ACOI 326 AB175837
Chlamydomonad sp. NDem9/21T-7w AY220575
Oogamochlamydinia
Carteria radiosa NIES-432 D86500
Carteria obtusa SAG 39.84 AF182818
Radicarteria
Spermatozopsis similis SAG B 1.85 X65557
Carteria olivieri UTEX LB 1032 U70596
Carteria crucifera D86501 NIES-421
Crucicarteria
Treubaria schmidlei SAG 36.83 U73474
Treubaria setigera SAG 37.83 U73475
Trochiscia hystrix UTEX LB 606 AF277651
Cylindrocapsa geminella SAG 3.87 U73471
Elakatothrix viridis Loras College CH30 AY008844
Treubarinia
Coelastrella saipanensis AB055800
Neochloris vigenis M74496
Pediastrum duplex M62997
Neocystis brevis CCALA 393 JQ920362
Lobosphaera incisa SAG 2007 AY762602
Parietochloris cohaerens UTEX 1707 EU878372
Chlorella sorokiniana KJ149805
Jenufa perforata CAUP H 8101 HM563743
Golenkinia longispicula SAG 73.80 JN968588
Outgroup
0.03
