# Supplementary figures and images for: Oogamochlamys kurilensis sp. nov. (Chlorophyta, Volvocales) from the Soils of Iturup Island (Sakhalin Region, Russia)
Source: Plants (Basel). 2023 Sep 22;12(19):3350. doi: 10.3390/plants12193350 (PMC10574126; doi:10.3390/plants12193350)

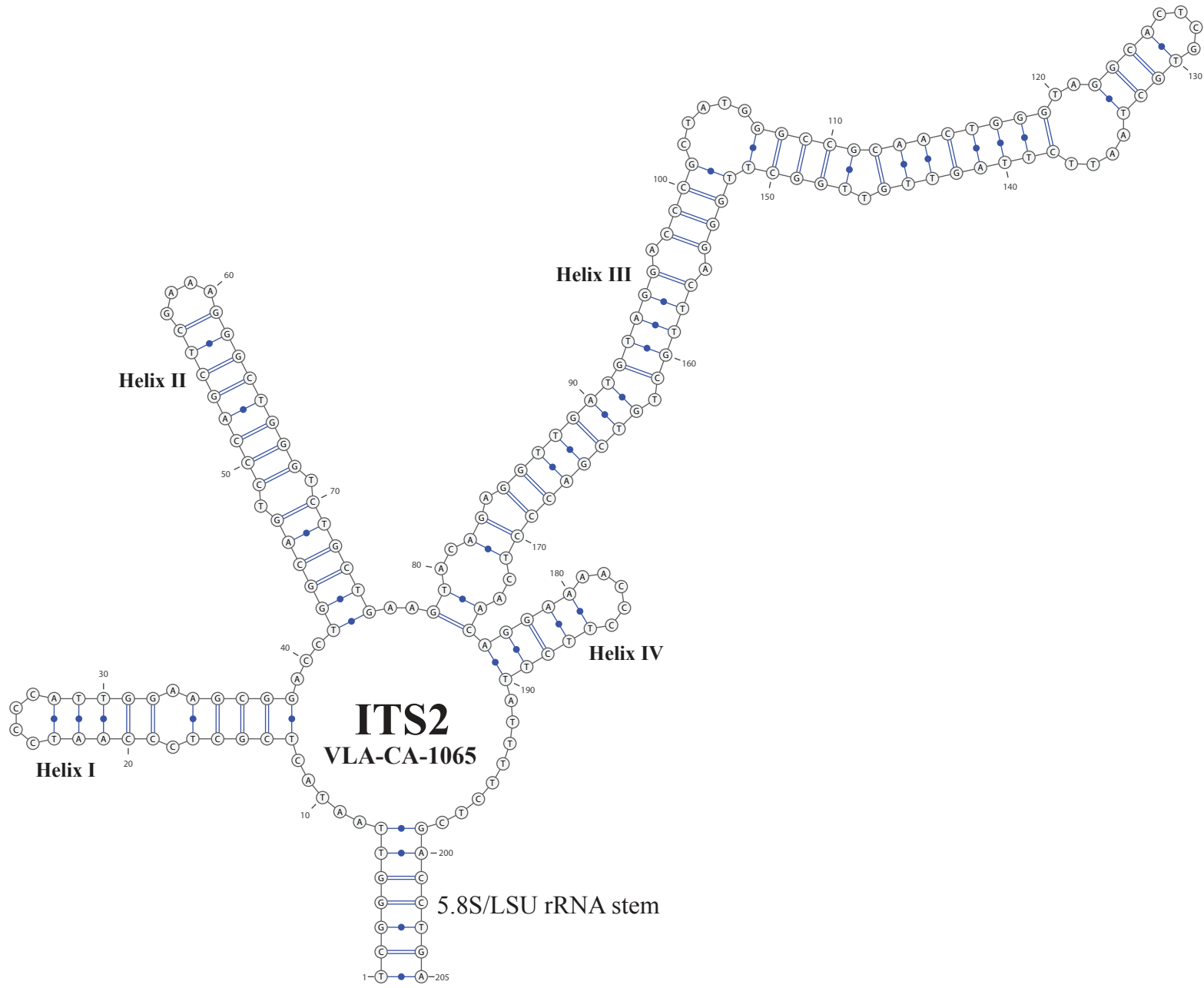

Supplement: Supplementary file 1 [file plants-12-03350-s001.zip › Figure S2.pdf]
